# Low-temperature catalytic oxidative coupling of methane in an electric field over a Ce–W–O catalyst system

**DOI:** 10.1038/srep25154

**Published:** 2016-04-27

**Authors:** Kei Sugiura, Shuhei Ogo, Kousei Iwasaki, Tomohiro Yabe, Yasushi Sekine

**Affiliations:** 1 Department of Applied Chemistry, Waseda University, 3-4-1, Okubo, Shinjuku, Tokyo, 169-8555 Japan

## Abstract

We examined oxidative coupling of methane (OCM) over various Ce–W–O catalysts at 423 K in an electric field. Ce_2_(WO_4_)_3_/CeO_2_ catalyst showed high OCM activity. In a periodic operation test over Ce_2_(WO_4_)_3_/CeO_2_ catalyst, C_2_ selectivity exceeded 60% during three redox cycles. However, Ce_2_(WO_4_)_3_/CeO_2_ catalyst without the electric field showed low activity, even at 1073 K: CH_4_ Conv., 6.0%; C_2_ Sel., 2.1%. A synergetic effect between the Ce_2_(WO_4_)_3_ structure and electric field created the reactive oxygen species for selective oxidation of methane. Results of XAFS, *in-situ* Raman and periodic operation tests demonstrated that OCM occurred as the lattice oxygen in Ce_2_(WO_4_)_3_ (short W–O bonds in distorted WO_4_ unit) was consumed. The consumed oxygen was reproduced by a redox mechanism in the electric field.

Natural gas is being discovered in many countries around the world. The United States and China have recently started to extract large amounts of shale gas. Nevertheless, the state of natural gas, especially methane, at room temperature and atmospheric pressures is gaseous. Therefore, it is transported using gas pipelines or LNG systems. Small and medium-sized natural gas fields have difficulty using such methods. Therefore, efficient conversion of methane to valuable chemicals and fuels is necessary for such cases. Methane conversion processes are categorized into two methods: selective oxidation of methane to useful hydrocarbons or oxygenates, and production of syngas by steam reforming, dry reforming, or partial oxidation of methane. We specifically examined direct catalytic methane conversion to C_2_ hydrocarbons by oxidative coupling of methane (OCM)^1^,^2^,^3^,^4^,^5^. The formula can be described as presented below (eq. 1).


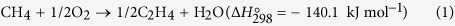


Because of its stable tetrahedral structure, methane activation requires temperatures higher than 973 K. Furthermore, the reactivity of ethylene is higher than that of methane. Consequently, C_2_ selectivity decreases because gas-phase non-selective and sequential oxidation with oxygen to form CO and CO_2_ is unavoidable at such high temperatures. Therefore, it is extremely difficult to obtain high C_2_ yield with OCM.

To resolve the difficulties described above, we adopted a non-conventional catalytic system, a catalytic reaction in an electric field, in anticipation of methane activation at low temperatures. Results show that various low-temperature catalytic reactions such as methane steam reforming^6^,^7^,^8^,^9^,^10^ can proceed in the electric field. We also reported that OCM proceeded at a low temperature (423 K) in the electric field over Sr-La_2_O_3_ (Sr/La = 1/20) catalyst^11^,^12^. Although the Sr-La_2_O_3_ catalyst showed high C_2_ selectivity (49.0%), CH_4_ conversion remained low (8.9%) in the electric field with imposition of 3.0 mA of current. Further catalyst development is necessary for OCM in an electric field at low temperatures.

As described in this paper, we specifically examined Ce–W–O system oxide catalysts, including Keggin-type heteropoly acids (HPAs) as catalysts, for improving OCM activity in the electric field. HPAs are inorganic metal-oxide anion clusters having multi-electronic redox properties^13^,^14^. Keggin-type heteropolytungstates are polyoxotungstates containing one central heteroatom X surrounded by 12 condensed W–O octahedra to form [XW_12_O_40_]^*n*−^ (X: P (*n* = 3), Si (*n* = 4), etc.). Keggin-type HPAs and the substituted Keggin-type HPAs show unique redox and catalytic properties^15^,^16^,^17^,^18^,^19^,^20^,^21^,^22^,^23^. Many studies of the partial oxidation of methane using HPAs have been conducted using these properties of HPAs. J. B. Moffat *et al.*^24^,^25^,^26^,^27^ reported the partial oxidation of methane into CO, HCHO, and CH_3_OH (oxidant: O_2_ and N_2_O) over HPAs/SiO_2_, but the conversion of methane was low (approx. 5%, 843 K). Mizuno *et al.*^28^,^29^,^30^,^31^ reported selective partial oxidation of propane with O_2_ over Cs_2.5_Fe_0.08_H_1.26_PVMo_11_O_40_. The yield of acrylic acid was 13% at 653 K. For the partial oxidation of methane, the conversion of methane was 0.2%; the catalyst showed almost no activity. Mizuno *et al.* concluded that the order of C–H bond strength is C_3_H_8_ < C_2_H_6_ < CH_4_ and that the difference in the activity for lower alkane conversions is attributable to the C–H bond strength.

Based on the discussion presented above, we investigated application of the electric field to Ce–W–O system catalysts including HPAs for low-temperature methane conversion. Important findings included the following: 1) 40 wt%TBA-PW_12_O_40_/CeO_2_ catalyst showed high OCM activity only in the electric field. The structure was changed to Ce_2_(WO_4_)_3_/CeO_2_. 2) The Ce_2_(WO_4_)_3_ structure, which functioned as an active site structure for selective oxidation of methane, worked only in an electric field. 3) Short W–O bonds of the distorted WO_4_ unit in Ce_2_(WO_4_)_3_ was an active site in the electric field; OCM occurred by a redox mechanism.

## Results and Discussion

### Activity tests over TBA-HPAs/CeO_2_ catalysts

First, catalytic oxidative coupling of methane (OCM) over various Ce–W–O oxide system catalysts including 40 wt%TBA-PW_12_O_40_/CeO_2_ (TBA: tetrabutylammonium) without an electric field was conducted at 573–1073 K (Supplementary Information Table S1). TBA-PW_12_O_40_/CeO_2_ catalyst showed low OCM activity (CH_4_ Conv., 5.0%; C_2_ Sel., 3.5%) without the electric field, even at the high temperatures of 1073 K.

Next, we evaluated the effects of an electric field on OCM activity over various catalysts including TBA-PW_12_O_40_/CeO_2_ catalyst (Supplementary Information Tables S2 and S3). TBA-PW_12_O_40_/CeO_2_ catalyst showed high OCM activity (CH_4_ Conv., 52.8%; C_2_ Sel., 32.0%; C_2_ Yield, 16.9% at imposed current 7.0 mA) in the electric field. Bare CeO_2_ catalyst showed high CH_4_ conversion (28.2%), but CeO_2_ contributed to complete oxidation of methane to produce CO_2_ with 98% selectivity in the electric field. Results show that the OCM activity of TBA-PW_12_O_40_/CeO_2_ was derived from the supported TBA-PW_12_O_40_. However, bare TBA-PW_12_O_40_ was unsuitable for imposing the electric field because of its electric conductivity.

As-made and after-reaction samples were characterized with Raman, XRD, and FT-IR (Supplementary Information Figs S1–S3). As-made samples were attributed to TBA-PW_12_O_40_ and CeO_2_. After reaction, the peaks corresponding to TBA-PW_12_O_40_ disappeared. New peaks, which were attributable to WO_3_ and Ce_2_(WO_4_)_3_, appeared. Therefore, it is likely that these oxides contribute to OCM activity.

To confirm the OCM activity for these oxides without an electric field, OCM over TBA-PW_12_O_40_/CeO_2_ catalyst at 573–1073 K without an electric field was conducted after reaction with an electric field (423 K, 3.0 mA) to form the oxides described above (Supplementary Information Fig. S4). With an electric field, TBA-PW_12_O_40_/CeO_2_ catalyst showed the following: CH_4_ Conv., 14.1%; C_2_ Sel., 44.4%. After turning off the electric field, CH_4_ and O_2_ conversion and C_2_ selectivity decreased dramatically at 1073 K: CH_4_ Conv., 2.1%; C_2_ Sel., 5.0%. Results show that the formed oxides can produce reactive oxygen species and activate methane only when an electric field is applied.

### Clarification of the active site structure

**Figure 1:**
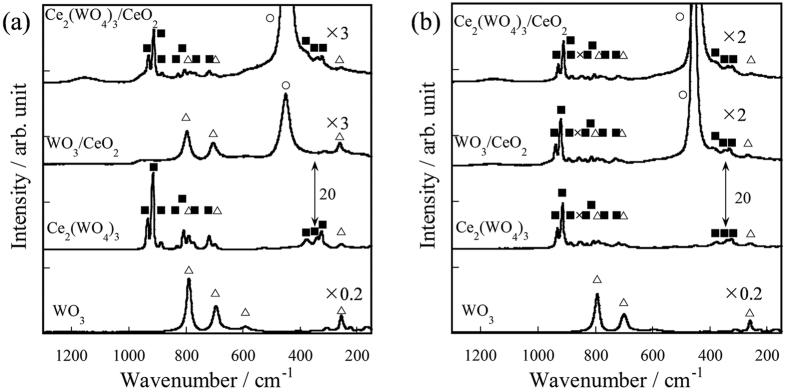
Raman spectra of various oxide catalysts: **(a)** as-made; **(b)** after reaction with electric field; ○, CeO_2_; Δ, WO_3_; ■, Ce_2_(WO_4_)_3_; ×, unidentified.

As described in the section above, the supported TBA-PW_12_O_40_ on CeO_2_ was converted to Ce_2_(WO_4_)_3_ and WO_3_ during OCM with an electric field. The formed oxides might play an important role for OCM with an electric field. Therefore, OCM activities over Ce_2_(WO_4_)_3_/CeO_2_, WO_3_/CeO_2_, Ce_2_(WO_4_)_3_, and WO_3_ catalysts were investigated in the electric field. Structures of the supported and the unsupported oxide catalysts were confirmed using Raman and XRD measurements (Fig. 1(a) and Supplementary Information Fig. S5(a)). Results of activity tests over these oxides at 423 K with an electric field are presented in Table 1, which shows that Ce_2_(WO_4_)_3_/CeO_2_, WO_3_/CeO_2_ and Ce_2_(WO_4_)_3_ showed OCM activity, although WO_3_ showed no OCM activity. Actually, Ce_2_(WO_4_)_3_/CeO_2_ showed the following for *T*_tc_ of 649 K: CH_4_ Conv., 13.6%; C_2_ Sel., 39.0%. For *T*_tc_ of 634 K, WO_3_/CeO_2_ showed the following: CH_4_ Conv., 14.3%; C_2_ Sel., 32.4%. Also, Ce_2_(WO_4_)_3_ showed the following for *T*_tc_ of 659 K: CH_4_ Conv., 9.7%; C_2_ Sel.: 41.2%. Additionally, WO_3_ catalyst showed no OCM activity even at almost the same temperature (around 650 K) or at almost the same input power (around 2.5 W) as the other catalysts (Supplementary Information Tables S4 and S5). Ce_2_(WO_4_)_3_/CeO_2_ catalyst showed the higher OCM activity than TBA-PW_12_/CeO_2_ catalyst at almost the same input power (2.6–2.7 W) (Supplementary Information Table S4).

The structures of Ce_2_(WO_4_)_3_/CeO_2_, WO_3_/CeO_2_, Ce_2_(WO_4_)_3_, and WO_3_ catalysts before and after reaction with electric field were analyzed using Raman spectroscopy and XRD (Fig. 1(b) and Supplementary Information Fig. S5(b)). As shown in Fig. 1(b), the structures of Ce_2_(WO_4_)_3_/CeO_2_, Ce_2_(WO_4_)_3_, and WO_3_ after reaction with the electric field were not markedly different from as-made. However, the spectrum of WO_3_/CeO_2_ after reaction with an electric field differed considerably from as-made. The Raman spectrum showed that W species in WO_3_/CeO_2_ changed to Ce_2_(WO_4_)_3_ from WO_3_ after reaction in the electric field. Supplementary Information Fig. S5 (XRD patterns) presents similar results. It is conceivable that the formation of Ce_2_(WO_4_)_3_ proceeded as a solid–solid reaction between CeO_2_ and WO_3_ in the reaction with an electric field^32^. In light of the activity test over WO_3_/CeO_2_, one can infer that the Ce_2_(WO_4_)_3_ formed on WO_3_/CeO_2_ activated methane and that it showed OCM activity. Therefore, activity tests and characterizations demonstrated that the active site structure for OCM with the electric field was Ce_2_(WO_4_)_3_. Moreover, the active site structure, Ce_2_(WO_4_)_3_, showed stable OCM activity for at least one hour (Supplementary Information Fig. S6).

### Contribution of the active site to OCM activity

To elucidate the contribution of the active site to OCM activity, OCM at 573–1073 K over Ce_2_(WO_4_)_3_/CeO_2_ catalyst was conducted without an electric field. Results of activity tests (573–1073 K) are presented in Table 2. In the reaction at 1073 K without the electric field, the catalyst showed results (CH_4_ Conv., 6.0%; C_2_ Sel., 2.1%) that were much lower than those in the reaction with the electric field at external temperature of 423 K (*T*_tc_ measured catalyst bed temperature by thermocouple: 649 K): CH_4_ Conv., 13.6%; C_2_ Sel., 39.0%. These results demonstrate that Ce_2_(WO_4_)_3_/CeO_2_ catalyst can produce reactive oxygen species and activate methane only when an electric field is applied.

In the reaction with an electric field, the catalyst bed temperature increased by Joule heating. Therefore, to elucidate the influence of Joule heating on OCM in the gas phase, the temperature dependency of OCM in the electric field was evaluated. Table 3 presents the activities of OCM over Ce_2_(WO_4_)_3_/CeO_2_ at 423, 673, and 873 K with the electric field (3.0 mA). C_2_ selectivity and C_2_ yield decreased in association with increasing furnace temperature. This reduction caused by combustion of C_2_ species with O_2_ in gas phase because O_2_ conversion increased in proportion to increasing temperature. Therefore, OCM in the gas phase is not promoted by Joule heating from the electric field. The effect of Joule heating by an electric field is unimportant in the system. In other words, because the catalyst was able to activate methane at a low gas-phase temperature, C_2_ selectivity was high in the OCM with the electric field at low temperature. The same trend was obtained in the case of electric power fixing to normalize the electric factor (Supplementary Information Table S6).

**Figure 2:**
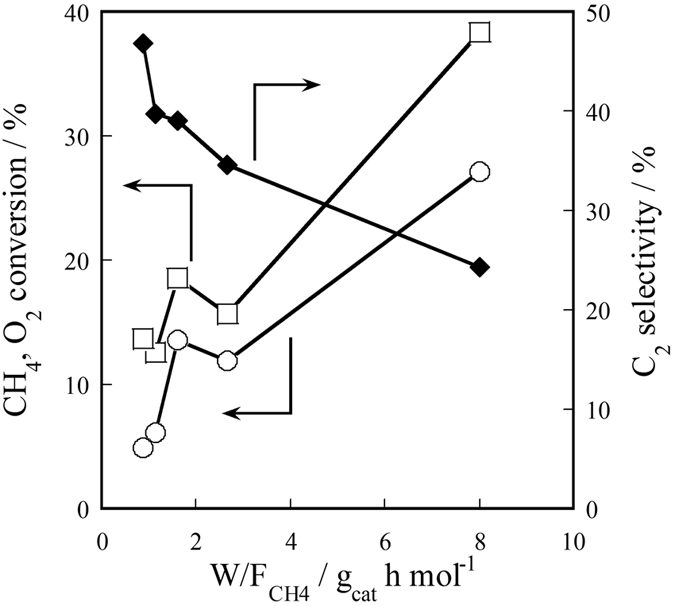
Influence of W/F_CH4_ on catalytic activity for OCM over Ce_2_(WO_4_)_3_/CeO_2_ in the electric field (423 K, 3.0 mA): CH_4_/O_2_ = 1.67; ◯, CH_4_ Conv.; □, O_2_ Conv.; ♦, C_2_ Sel.

Next, to clarify the reaction mechanism, the influence of contact time (W/F_CH4_) on OCM activity over Ce_2_(WO_4_)_3_/CeO_2_ in the electric field was investigated. As presented in Fig. 2, CH_4_ conversion and O_2_ conversion increased and C_2_ selectivity decreased concomitantly with increasing contact time. The decrease of C_2_ selectivity resulted from combustion of C_2_ hydrocarbons with O_2_ in gas phase.

**Figure 3:**
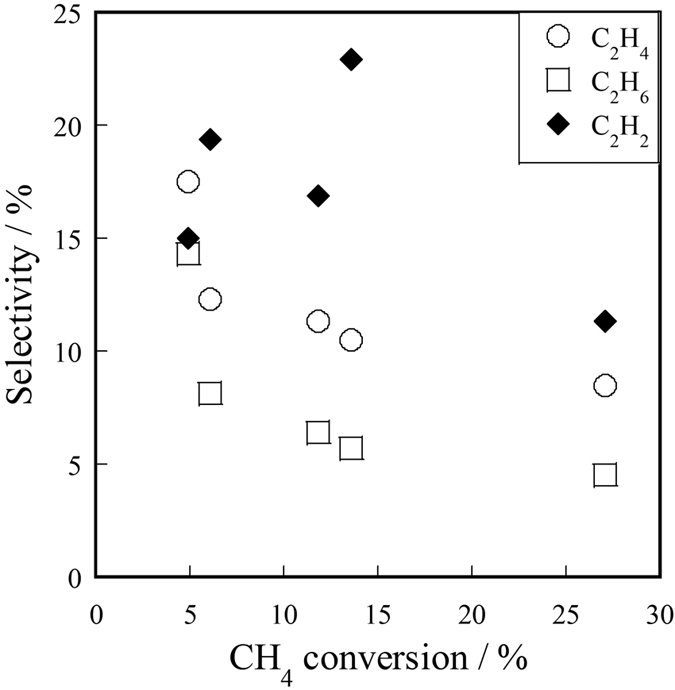
Relation between CH_4_ conversion and C_2_H_4_, C_2_H_6_, C_2_H_2_ selectivity over Ce_2_(WO_4_)_3_/CeO_2_ in the electric field (423 K, 3.0 mA): CH_4_/O_2_ = 1.67; ◯, C_2_H_4_; □, C_2_H_6_; ♦, C_2_H_2_.

Figure 3 shows the relation between CH_4_ conversion and C_2_H_4_, C_2_H_6_, and C_2_H_2_ selectivity over Ce_2_(WO_4_)_3_/CeO_2_ in the electric field (423 K, 3.0 mA). As shown in Fig. 3, as CH_4_ conversion approaches 0%, C_2_H_4_ and C_2_H_6_ selectivity increase and C_2_H_2_ selectivity decreases. In the range of very low CH_4_ conversion, production of C_2_H_4_ and C_2_H_6_ were the main reactions. One can infer that methyl radical and carbene were produced on the catalyst surface in the electric field. Accordingly, Ce_2_(WO_4_)_3_ can extract one or two H atoms from CH_4_ and produced methyl radical and carbene. However, C_2_H_2_ selectivity increased concomitantly with increased CH_4_ conversion up to 15% and then decreased. These results suggest that C_2_H_2_ was generated through oxidative dehydrogenation of C_2_H_4_ or through coupling of CH species, which was formed from CH_4_ with electric energy^33^; then it was oxidized to CO and CO_2_.

To confirm that reactive oxygen species are formed on the catalyst surface in the electric field, the periodic operation test at 473 K over Ce_2_(WO_4_)_3_/CeO_2_ catalyst was conducted with the electric field. Results of activity tests are presented in Table 4. The results were the activities at 2 min after from methane+Ar supply. Table 4 shows that the C_2_ selectivity was higher than 60% and that it was maintained during three cycles. When increasing the CH_4_ conversion by increasing the contact time, C_2_ selectivity was higher than 65% and was maintained during three cycles. However, in the periodic operation test without an electric field at 1073 K, C_2_ selectivity was very low; CO and CO_2_ were mainly formed (Supplementary Information Table S7). These results demonstrate that reactive oxygen species suitable for OCM were formed on the catalyst surface in the electric field. The synergic effect of Ce_2_(WO_4_)_3_ and electric field created the reactive oxygen species for selective oxidation of methane and activated methane because the active site structure for OCM with the electric field was Ce_2_(WO_4_)_3_.

### *In-situ* Raman over Ce_2_(WO_4_)_3_/CeO_2_ with and without electric field

**Figure 4:**
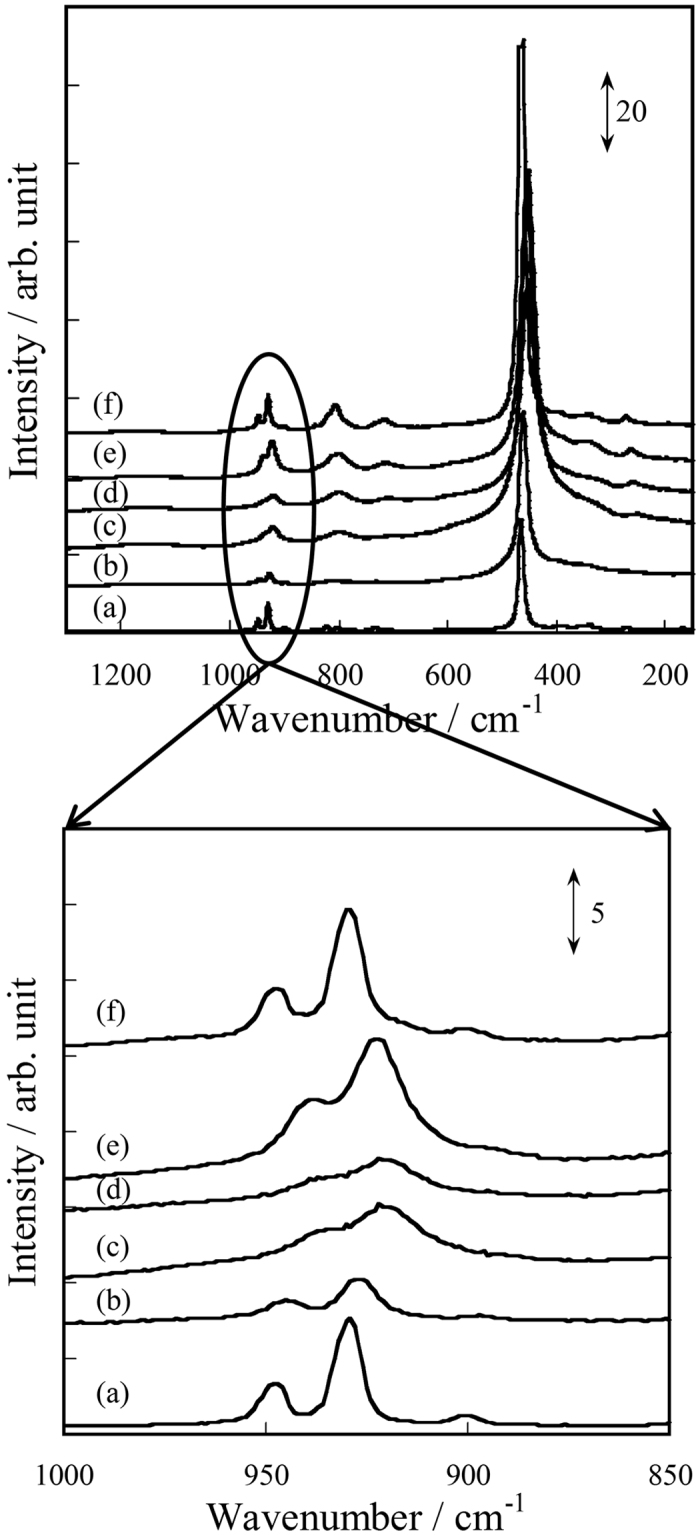
Raman spectra of Ce_2_(WO_4_)_3_/CeO_2_ with and without electric field: (a) inert at room temperature, (b) without EF in air at 603–703 K, (c) 6.0 mA in air (0.69 kV), (d) 6.0 mA in CH_4_ (0.73 kV), (e) 6.0 mA in CH_4_ + O_2_ (0.67 kV), and (f) without EF after (e).

Many researchers have reported that Na_2_WO_4_-Mn_2_O_3_/SiO_2_ catalyst has high OCM activity^2^,^34^. A short W–O bond in the distorted WO_4_ unit is proposed as the active site of Na_2_WO_4_-Mn_2_O_3_/SiO_2_. This W–O bond is observed at 927 cm^−1^ in Raman spectrum^35^. Specific examination of the Ce_2_(WO_4_)_3_ structure reveals that it has WO_4_ units of two kinds. Both are physically distorted. It is likely that W–O bonds in Ce_2_(WO_4_)_3_/CeO_2_ work as an active site for OCM in the electric field. Comparison of Ce_2_(WO_4_)_3_ with Na_2_WO_4_ reveals that Ce_2_(WO_4_)_3_ has a stable structure and short W–O bonds in the distorted WO_4_ unit, which are observed at 949 cm^−1^ and 931 cm^−1^ 32. We conducted *in-situ* Raman measurements over Ce_2_(WO_4_)_3_/CeO_2_ in the electric field to clarify the W–O bond behavior in the electric field. Figure 4 portrays Raman spectra with and without the electric field. The peak positions of the short W–O bonds in distorted WO_4_ unit are summarized in Supplementary Information Table S8. Figure 4 shows that the spectra of Ce_2_(WO_4_)_3_/CeO_2_ with an electric field (Fig. 4c–e) differed from inert (Fig. 4a) and the peak of W–O bonds shifted to a lower wavenumber and broadened. From Supplementary Information Table S8, the peak shift of W–O peaks in the electric field was about 7–10 cm^−1^. The shift and broadening of the W–O peaks were attributed to the electric field because the spectrum without electric field after imposing electric field in CH_4_ + O_2_ flow (Fig. 4f) was almost identical to that of the spectrum without an electric field under inert (Fig. 4a). These results suggest that the Ce_2_(WO_4_)_3_/CeO_2_ structure was distorted and that the W–O bonds were weakened by the electric field. It acted as the reactive oxygen suitable for selective oxidation of methane to C_2_ hydrocarbons. For comparison, the Raman spectrum over Ce_2_(WO_4_)_3_/CeO_2_ at 603–703 K without an electric field (Fig. 4b) was measured because the catalyst bed temperature was increased by Joule heating in the electric field. The shift of W–O peaks was about 2–3 cm^−1^, indicating a small effect of Joule heat from the electric field on W–O bond activation. These results imply that W–O bond activation is attributable mainly to the electric field and that the W–O bond activated by the electric field functioned as an active site for OCM in the electric field.

Next, XAFS spectra were recorded. The results are presented in Supplementary Information Figs S7 and S8 and Table S9. Regarding the coordination number of W–O bonds in Ce_2_(WO_4_)_3_ from XAFS measurements, the catalyst after 1 cycle in periodic operation test in the electric field showed a smaller coordination number than in the as-made material and after O_2_ supply in the periodic operation test in the electric field. This result supports our inference that surface oxygen of Ce_2_(WO_4_)_3_ activated by the electric field was consumed by methane. Therefore, results of XAFS, *in-situ* Raman, and periodic operation tests demonstrated that OCM which occurred as lattice oxygen in Ce_2_(WO_4_)_3_ (short W–O bonds in distorted WO_4_ unit) was consumed and reproduced by the redox mechanism. A possible reaction mechanism is described in Supplementary Information Fig. S9. In addition, redox reaction of Ce cation (Ce^3+^ ↔ Ce^4+^ + e^‒^) might be also responsible for the OCM reaction.

## Conclusion

Oxidative coupling of methane (OCM) over 40 wt%TBA-PW_12_O_40_/CeO_2_ in the electric field was conducted. The catalyst showed high OCM activity, although the catalyst showed extremely low OCM activity, even at the high temperature of 1073 K without an electric field. After reaction with the electric field, Raman spectra confirmed that the structure of TBA-PW_12_O_40_/CeO_2_ catalyst was changed to Ce_2_(WO_4_)_3_/CeO_2_.

Then, OCM activities over Ce_2_(WO_4_)_3_/CeO_2_, WO_3_/CeO_2_, Ce_2_(WO_4_)_3_, and WO_3_ catalysts in an electric field (3.0 mA) were investigated to clarify the structure of the active site. Ce_2_(WO_4_)_3_/CeO_2_, WO_3_/CeO_2_ and Ce_2_(WO_4_)_3_ catalysts showed OCM activity; WO_3_ showed no OCM activity. After reaction with the electric field, Raman spectra showed that W species in WO_3_/CeO_2_ changed to Ce_2_(WO_4_)_3_ from WO_3_. Therefore, the active site structure for OCM with the electric field was Ce_2_(WO_4_)_3_.

Also, Ce_2_(WO_4_)_3_/CeO_2_ catalyst showed extremely low OCM activity at 1073 K. In the periodic operation test over Ce_2_(WO_4_)_3_/CeO_2_ catalyst, C_2_ selectivity was higher than 60% and was maintained during three cycles. Therefore, synergic effects of Ce_2_(WO_4_)_3_ and the electric field created the reactive oxygen species suitable for selective oxidation of methane, activated methane, and progressed OCM only when an electric field was applied.

Results of XAFS, *in-situ* Raman and periodic operation test demonstrated that OCM occurred using the lattice oxygen of Ce_2_(WO_4_)_3_ (short W–O bonds in distorted WO_4_ unit), which were consumed and reproduced by the redox mechanism in the electric field.

## Methods

### Catalyst preparation

Tetrabutylammonium (TBA) salt of Keggin-type HPAs (denoted as TBA-HPAs), such as (TBA)_*n*_[PW_12-*x*_V_*x*_O_40_] (*x* = 0, 1, 2; *n* = 3, 4, 5), were prepared according to the published procedure with some modifications^36^,^37^,^38^,^39^. They were analyzed using IR spectroscopy (*see* Supplementary Information). As a reference catalyst, WO_3_ (Kanto Chemical Co. Inc.) was used as supplied. All other chemicals were reagent-grade; they were used as supplied.

Keggin-type TBA-HPAs supported on CeO_2_ (JRC-CEO-1) catalysts were prepared by impregnation with acetone as the impregnation solvent. The loading amount of TBA-HPAs was 40 wt%. First, acetone (30 mL) and CeO_2_ (0.6 g) were added to a 300 mL eggplant flask and were stirred for 2 h using a rotary evaporator. Subsequently, TBA-HPAs (0.4 g) dissolved into acetone (10 mL) were added to the flask and were stirred for 2 h again. The resulting suspension was dried. Then the resulting solid was dried overnight at 393 K.

WO_3_/CeO_2_ catalyst containing 11.9 wt% W was prepared using impregnation with water as the impregnation solvent, as described in a previous report^32^. An ammonium metatungstate hydrate ((NH_4_)_6_H_2_W_12_O_40_·H_2_O) was used as a precursor. After impregnation, the resulting suspension was dried. Then the resulting solid was dried at 393 K overnight, followed by calcination for 3 h in air at 773 K under a ramping rate of 0.5 K min^−1^.

Ce_2_(WO_4_)_3_/CeO_2_ catalyst containing 11.9 wt% W was prepared by impregnating CeO_2_ with an aqueous solution of ammonium metatungstate hydrate using a similar method to that for WO_3_/CeO_2_, except that the calcination temperature was 1173 K^32^. As a reference, unsupported Ce_2_(WO_4_)_3_ was prepared using a complex method combining ethylenediamine tetraacetic acid and citrate ions, as described in previous reports^40^,^41^.

### Activity test

Catalytic activity tests were conducted with a fixed bed flow-type reactor equipped with a quartz tube (4.0 mm i.d.). A schematic image of the reaction system is presented in Supplementary Information Fig. S10. The catalyst was sieved into 355–500 μm. Then 100 mg of it was charged in the reactor. The reactant feed gases were methane, oxygen, and Ar (CH_4_: O_2_: Ar = 25: 15: 60, total flow rate: 100 SCCM). The effect of contact time (W/F_CH4_) was investigated by changing the total flow rate. The standard W/F_CH4_ was 1.6 g_cat_ h mol^−1^. For the reaction in the electric field, two stainless steel electrodes (2.0 mm o.d.) were inserted contiguously into the catalyst bed in the reactor. The electric field was controlled using a constant current (3, 5, or 7 mA) with a DC power supply. The imposed voltage depended on the electric properties of the catalyst. Current and voltage profiles were measured using an oscilloscope (TDS 3052B; Tektronix Inc.). The reactor temperature was set to 423 K to avoid the condensation of water produced by the reactions, except for reactions that used no electric field. Product gases after passing a cold trap were analyzed using GC-FID (GC-14B; Shimadzu Corp.) with a Porapak N packed column and methanizer (Ru/Al_2_O_3_ catalyst), and using a GC-TCD (GC-2014; Shimadzu Corp.) with a molecular sieve 5A packed column. The respective calculation formulae for conversion, C_2_ yield, C_2_ selectivity, and Faradaic number in this study are shown below (eqs 2–6).


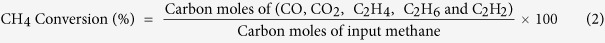



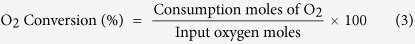



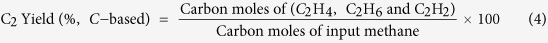



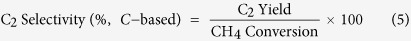



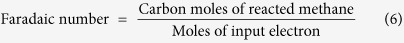


A periodic operation test was conducted to elucidate surface active species on the catalyst in the following steps. In the first step, oxygen and Ar were supplied to the reactor with an electric field for 10 min for oxidation of the catalyst surface. For the second step, residual oxygen in the gas phase of the reactor was removed with Ar purge for 5 min. For the third step, methane and Ar were supplied to the reactor with an electric field for 12 min to evaluate the oxidation catalysis of the surface oxygen species on the catalyst. As the final step, Ar purge was conducted for 20 min to remove all residual gases. The steps described above were repeated for three cycles. Product gases were analyzed at 5 min after oxygen+Ar supply, and at 2 and 12 min after methane +Ar supply (CO_x_ and desorbed CH_4_ were detected at 5 min after from oxygen+Ar supply and no products were detected at 12 min after from methane + Ar supply). Gas flow was O_2_: Ar = 1: 10, total 55 mL min^−1^ (for oxidation of the catalyst surface) and CH_4_: Ar = 1: 10 or 1: 2, total 55 or 75 mL min^−1^ (for oxidation of supplied methane by surface oxygen species). The reactor temperature was fixed at 473 K. The imposed current was set at 3.0 or 7.0 mA.

### Characterization of catalyst

FT–IR spectra were recorded on a spectrometer (FT-IR/6200; Jasco Corp.) using a KBr pelletizing method. Raman spectra were recorded on a Raman spectrometer (excitation line λ = 532 nm, NRS-1000; Jasco Corp.). The crystalline structure was characterized using powder X-ray diffraction (XRD, RINT-Ultima III; Rigaku Corp.) operating at 40 kV and 40 mA with Cu-Kα radiation. The specific surface area of the catalyst was measured using N_2_ adsorption using the BET method (Gemini VII; Micromeritics Instrument Corp.) after pre-treatment at 473 K in N_2_ atmosphere for 2 h. Results of BET measurements are presented in Supplementary Information Table S10. W L_3_-edge X-ray absorption fine structure (XAFS) spectra were recorded on BL14B2 in SPring-8 (Hyogo, Japan). Catalysts treated in the reaction condition were ground into powder and were pressed into pellets. Then, pellets were packed into gas-barrier bags. The pellets were diluted with BN to adjust for XAFS measurement. EXAFS analysis and curve fitting were performed using software (Athena ver. 0.8.056; Artemis ver. 0.8.012).

### *In-situ* Raman

*In-situ* Raman measurements in the electric field were conducted using a Raman spectrometer with a hand-made glass reactor and gold wire electrodes (see Supplementary Information Fig. S11). The reactant feed gases were supplied with canned standard gases (CH_4_(0.995%) + Ar and/or O_2_(99.9%)). The electric field was imposed using a constant current at 6.0 mA.

Raman spectra for catalyst heated at reaction temperature (603–703 K) without an electric field were also observed to elucidate the effect of Joule heating on the catalyst structure. A Ni-Cr wire was inserted into the sample for heating by resistance heating.

## Additional Information

**How to cite this article**: Sugiura, K. *et al.* Low-temperature catalytic oxidative coupling of methane in an electric field over a Ce-W-O catalyst system. *Sci. Rep.* **6**, 25154; doi: 10.1038/srep25154 (2016).

## Supplementary Material

Supplementary Information

## Figures and Tables

**Table 1:** Catalytic activities over various oxide catalysts in the electric field^a^.

Catalysts	T_tc_^b^/K	Voltage/KV	CH_4_ Conv./%	O_2_ Conv./%	C_2_ Sel./%	C_2_ Yield/%	Field intensity/V mm^−1^	Faradiac number/−
TBA-PW_12_/CeO_2_	689	1.3	14.9	20.6	43.4	6.4	260	83.3
Ce_2_(WO_4_)_3_/CeO_2_	649	0.9	13.6	18.5	39.0	5.3	225	73.9
WO_3_/CeO_2_	634	0.8	14.3	20.8	32.4	4.6	145	74.0
Ce_2_(WO_4_)_3_	659	0.7	9.7	11.6	41.2	4.0	189	53.3
WO_3_	484	0.2	0.0	2.5	0.0	0.0	54	0.2

^a^Feed gas, CH_4_:O_2_:Ar = 25:15:60 SCCM; input current, 3.0 mA; catalyst weight, 100 mg; furnace temperature, 423 K.

^b^Catalyst bed temperature measured using a thermocouple.

**Table 2:** Effect of reaction field on catalytic activity over Ce_2_(WO_4_)_3_/CeO_2_ with and without an electric field.

Conditions	External temp./K	CH_4_ Conv./%	O_2_ Conv./%	C_2_ Sel./%	C_2_ Yield/%
with EF (3.0 mA)^a^	423 (649)^c^	13.6	18.5	39.0	5.3
without EF^b^	573	0.0	0.5	0.0	0.0
	673	0.0	0.7	0.0	0.0
	773	0.0	1.6	0.0	0.0
	873	0.1	2.5	0.0	0.0
	973	0.5	2.1	0.0	0.0
	1073	6.0	14.8	2.1	0.1

^a^Feed gas, CH_4_:O_2_:Ar = 25:15:60 SCCM; input current, 3.0 mA; catalyst weight, 100 mg; furnace temperature, 423 K.

^b^Feed gas, CH_4_:O_2_:Ar = 25:15:60 SCCM; catalyst weight, 100 mg; furnace temperature, 573–1073 K.

^c^Catalyst bed temperature measured using a thermocouple.

**Table 3:** Temperature dependency over Ce_2_(WO_4_)_3_/CeO_2_ in the electric field^a^.

Furnace temp./K	*T*_tc_^b^/K	Voltage/kV	CH_4_ Conv./%	O_2_ Conv./%	C_2_ Sel./%	C_2_ Yield/%	Field intensity/V mm^−1^
423	649	0.9	13.6	18.5	39.0	5.3	225
673	793	0.6	7.5	25.5	38.4	2.9	146
873	931	0.3	4.7	31.5	21.4	1.0	71

^a^Feed gas, CH_4_:O_2_:Ar = 25:15:60 SCCM; input current, 3.0 mA; catalyst weight, 100 mg; furnace temperature, 423, 673, 873 K.

^b^Catalyst bed temperature measured using a thermocouple.

**Table 4:** Results of periodic operation test (after 2 min from CH_4_ supply) over Ce_2_(WO_4_)_3_/CeO_2_ in the electric field.

CH_4_ flow rate/SCCM	Cycle number/-	*T*_tc_^c^/K	Voltage/V	CH_4_ Conv./%	C_2_ Sel./%	CO_x_ Sel./%	C_2_ Yield/%	Field intensity/V mm^−1^
25^a^	1	577	0.2	0.02	60.0	40.0	0.01	54
	2	603	0.2	0.06	73.0	27.0	0.04	54
	3	593	0.2	0.08	63.2	36.8	0.05	54
5^b^	1	522	0.2	2.6	74.4	25.6	1.9	48
	2	520	0.3	1.4	67.4	32.6	0.9	71
	3	515	0.1	1.4	70.6	29.4	1.0	24

^a^Feed gas, O_2_:Ar = 5:50 SCCM, CH_4_:Ar = 25:50 SCCM; input current, 7.0 mA; catalyst weight, 100 mg; furnace temperature, 473 K.

^b^Feed gas, O_2_:Ar = 5:50 SCCM, CH_4_:Ar = 5:50 SCCM; input current, 3.0 mA; catalyst weight, 100 mg; furnace temperature, 473 K.

^c^Catalyst bed temperature measured using a thermocouple.
