# Supplementary material for: Low-temperature catalytic oxidative coupling of methane in an electric field over a Ce–W–O catalyst system
Source: Sci Rep. 2016 Apr 27;6:25154. doi: 10.1038/srep25154 (PMC4846874; doi:10.1038/srep25154)
Supplement: Supplementary Information [file srep25154-s1.pdf]

Supporting information

Low temperature catalytic oxidative coupling of methane in an electric field over Ce-W-O catalyst system

Kei Sugiura, Shuhei Ogo*, Kousei Iwasaki, Tomohiro Yabe, Yasushi Sekine

Department of Applied Chemistry, Waseda University, 3-4-1, Okubo, Shinjuku, Tokyo, 169-8555 Japan

*: Corresponding author: ogo@aoni.waseda.jp

Materials

A stock solution of 1.0 M of V(V) was prepared by dissolving 9.186 g of V_2O_5 and 6.194 g of NaOH in 100 mL of water (0.5 M V_2O_5/1.5M NaOH). Other reagents were of analytical grade and were used as received.

Preparations of tetrabutylammonium (TBA) salt of Keggin-type HPAs

Preparation of $(TBA)_3[PW_{12}O_{40}]$ (denoted as TBA-PW_{12})

An aqueous solutions of 1.0 M Na_2WO_4 (5 mL), conc. HCl (4.2 mL), and 1.0 M NaH_2PO_4 solution (0.5 mL) were poured into 100 mL vessel in the order, and diluted with 100 mL of water. The resultant solution was aged at 353 K for 2 days in static. After the resulting clear solution had been cooled to room temperature, three equivalents (0.484 g) of tetrabutylammonium (TBA) bromide, $[CH_3(CH_2)_3]_4NBr$, was added to solution. After solution stirred at room temperature for 30 min, the resultant white solid was filtered off, washed with distilled water (50 mL) and ethanol (20 mL), and dried at 393 K overnight.

IR(KBr): ν = 1079 (m), 975 (s), 895 (s), 814 (s) cm^{-1}.

Preparation of $(TBA)_4[PW_{11}V_1O_{40}]$ (denoted as TBA-$PW_{11}V_1$)

An aqueous solutions of 1.0 M Na_2WO_4 (5 mL), 0.5 M V_2O_5/1.5M NaOH (0.5 mL), conc. HCl (4.2 mL), and 1.0 M NaH_2PO_4 solution (0.5 mL) were poured into 100 mL vessel in the order, and diluted with 100 mL of water. The resultant solution was aged at 353 K for 2 days in static. After the resulting yellow solution had been cooled to room temperature, four equivalents (0.645 g) of tetrabutylammonium (TBA) bromide, $[CH_3(CH_2)_3]_4NBr$, was added to solution. After solution stirred at room temperature for

30 min, the resultant yellow solid was filtered off, washed with distilled water (50 mL) and ethanol (20 mL), and dried at 393 K overnight.

IR(KBr): ν = 1095 (w), 1069 (w), 962 (s), 889 (m), 808 (vs) cm^{-1}.

Preparation of $(TBA)_5[PW_{10}V_2O_{40}]$ (denoted as TBA-$PW_{10}V_2$)

An aqueous solutions of 1.0 M Na_2WO_4 (5 mL), 0.5 M V_2O_5/1.5M NaOH (2.0 mL), conc. HCl (4.2 mL), and 1.0 M NaH_2PO_4 solution (0.5 mL) were poured into 100 mL vessel in the order, and diluted with 100 mL of water. The resultant solution was aged at 353 K for 2 days in static. After the resulting orange solution had been cooled to room temperature, five equivalents (0.806 g) of tetrabutylammonium (TBA) bromide, $[CH_3(CH_2)_3]_4NBr$, was added to solution. After solution stirred at room temperature for 30 min, the resultant orange solid was filtered off, washed with distilled water (50 mL) and ethanol (20 mL), and dried at 393 K overnight.

IR(KBr): ν = 1095(w), 1063 (w), 960 (s), 889 (m), 808 (vs) cm^{-1}.

Table S1 Catalytic activities over 40 wt%TBA-$PW_{12}O_{40}$/CeO_2 in various temperatures without electric field[a]

Condition	External temp. / K	CH_4 Conv. / %	O_2 Conv. / %	C_2 Sel. / %	C_2 Yield / %
without EF	573	0.1	0.7	0.0	0.0
	673	0.1	2.1	0.0	0.0
	773	0.4	4.2	0.0	0.0
	873	1.3	3.8	0.0	0.0
	973	1.3	5.9	0.0	0.0
	1073	5.0	14.8	3.5	0.2

[a] Feed gas CH_4:O_2:Ar = 25:15:60 SCCM, catalyst weight: 100 mg, furnace temperature: 573-1073 K

Table S2 Effect of input current on catalytic activity over 40 wt%TBA-$PW_{12}O_{40}$/CeO_2 in the electric field[a]

Current / mA	T_{tc}[b] / K	Voltage / kV	CH_4 Conv. / %	O_2 Conv. / %	C_2 Sel. / %	C_2 Yield / %	Field intensity / V mm^{-1}	Faradaic number / -
3.0	689	1.3	14.9	20.6	43.4	6.4	260	83.3
5.0	772	0.8	32.5	40.1	40.0	13.0	160	109
7.0	863	0.7	52.8	63.3	32.0	16.9	140	127

[a] Feed gas CH_4:O_2:Ar = 25:15:60 SCCM, input current: 3.0-7.0 mA, catalyst weight: 100 mg, furnace temperature: 423 K

[b] Catalyst bed temperature measured by a thermocouple

Table S3 Catalytic activities over 40 wt%TBA-HPAs/CeO_2 in the electric field[a]

Catalysts	T_{tc}[b] / K	Voltage / kV	CH_4 Conv. / %	O_2 Conv. / %	C_2 Sel. / %	C_2 Yield / %	Field intensity / V mm^{-1}	Faradaic number / -
TBA-PW_{12}/CeO_2	772	0.8	32.5	40.1	40.0	13.0	160	109
TBA-$PW_{11}V_1$/CeO_2	754	0.8	26.4	34.7	31.8	8.4	136	83.4
TBA-$PW_{10}V_2$/CeO_2	708	0.7	25.5	34.2	31.4	8.0	130	81.1
CeO_2	675	0.1	28.2	100	0.2	0.1	20	93.1

[a] Feed gas CH_4:O_2:Ar = 25:15:60 SCCM, input current: 5.0 mA, catalyst weight: 100 mg, furnace temperature: 423 K

[b] Catalyst bed temperature measured by a thermocouple

TBA-HPAs: TBA-$PW_{12-x}V_xO_{40}$ (x = 0~2)

Table S4 Catalytic activities over various oxide catalysts in the electric field (power fixing)[a]

Catalysts	T_{tc}[b] / K	Current / mA	Power / W	CH_4 Conv. / %	O_2 Conv. / %	C_2 Sel. / %	C_2 Yield / %	Field intensity / V mm^{-1}	Faradaic number / -
TBA-PW_{12}/CeO_2	595	1.5	2.6	5.8	14.5	43.7	2.6	315	61.4
$Ce_2(WO_4)_3$/CeO_2	649	3.0	2.7	13.6	18.5	39.0	5.3	225	73.9
WO_3/CeO_2	634	3.0	2.4	14.3	20.8	32.4	4.6	145	74.0
$Ce_2(WO_4)_3$	659	3.0	2.1	9.7	11.6	41.2	4.0	189	53.3
WO_3	586	20.0	2.0	0.2	1.7	0.0	0.0	29	0.1

[a] Feed gas CH_4:O_2:Ar = 25:15:60 SCCM, input current: 1.5, 3.0, 20.0 mA, catalyst weight: 100 mg, furnace temperature: 423 K

[b] Catalyst bed temperature measured by a thermocouple

Table S5 Catalytic activities over various oxide catalysts in the electric field (catalyst bed temperature fixing)[a]

Catalysts	T_{tc}[c] / K	Voltage / kV	CH_4 Conv. / %	O_2 Conv. / %	C_2 Sel. / %	C_2 Yield / %	Field intensity / V mm^{-1}	Faradaic number / -
TBA-PW_{12}/CeO_2	689	1.3	14.9	20.6	43.4	6.4	260	83.3
$Ce_2(WO_4)_3$/CeO_2	649	0.9	13.6	18.5	39.0	5.3	225	73.9
WO_3/CeO_2	634	0.8	14.3	20.8	32.4	4.6	145	74.0
$Ce_2(WO_4)_3$	659	0.7	9.7	11.6	41.2	4.0	189	53.3
WO_3 [b]	684	0.1	0.0	14.3	0.0	0.0	29	0.0

[a] Feed gas CH_4:O_2:Ar = 25:15:60 SCCM, input current: 3.0 mA, catalyst weight: 100 mg, furnace temperature: 423 K ([b] 673 K)

[c] Catalyst bed temperature measured by a thermocouple

Table S6 Temperature dependency over $Ce_2(WO_4)_3$/CeO_2 in the electric field (power fixing)[a]

Furnace temp. / K	T_{tc}[b] / K	Current / mA	Voltage / kV	Power / W	CH_4 Conv. / %	O_2 Conv. / %	C_2 Sel. / %	C_2 Yield / %	Field intensity / V mm^{-1}
423	649	3.0	0.9	2.7	13.6	18.5	39.0	5.3	225
673	830	5.0	0.5	2.5	16.2	36.9	33.7	5.5	122
873	993	10.0	0.3	3.0	18.7	47.1	18.0	3.4	77

[a] Feed gas CH_4:O_2:Ar = 25:15:60 SCCM, input current: 3.0, 5.0, 10.0 mA, catalyst weight: 100 mg, furnace temperature: 423, 673, 873 K

[b] Catalyst bed temperature measured by a thermocouple

Table S7 Result of periodic operation test (after 2 and 12 min from CH_4 supply) over $Ce_2(WO_4)_3/CeO_2$ without electric field at 1073 K[a]

Cycle number / -	Time / min	CH_4 Conv. / %	C_2 Sel. / %	CO_x Sel. / %	C_2 Yield / %
1	2	0.17	11.6	88.4	0.02
	12	0.39	4.1	95.9	0.02
2	2	0.18	8.3	91.7	0.02
	12	0.37	4.5	95.5	0.02
3	2	0.20	8.6	91.4	0.02
	12	0.39	4.4	95.6	0.02
4	2	0.22	7.7	92.3	0.02
	12	0.40	4.3	95.7	0.02
5	2	0.23	8.0	92.0	0.02
	12	0.44	4.1	95.9	0.02

[a] Feed gas: O_2:Ar = 15:60 SCCM, CH_4:Ar = 25:60 SCCM, catalyst weight: 100 mg, furnace temperature: 1073 K

Table S8 Results of *in-situ* Raman over $Ce_2(WO_4)_3/CeO_2$ with and without electric field

Conditions		Voltage / kV	Wavenumber / cm^{-1} $Ce_2(WO_4)_3$ W^{I}-O	W^{II}-O
(a)	inert (RT)	-	948	929
(b)	without EF (air, 603-703 K)	-	945	927
(c)	with EF (air, 6.0 mA)	0.69	937	922
(d)	with EF (CH_4, 6.0 mA)	0.73	938	922
(e)	with EF (CH_4+O_2, 6.0 mA)	0.67	938	922
(f)	without EF after (e)	-	947	930

Table S9 Result of curve fitting over $Ce_2(WO_4)_3/CeO_2$ catalysts in various state[a]

Conditions	bond	CN	R / A	dE / eV	DW / A^2	R-factor
as-made	W-O	1.05	1.730	3.652	0.0040	0.047
	W-O		1.768			
	W-O		1.812			
	W-O		1.818			
	W-O		2.197			
after O_2 supply in periodic operation test	W-O	1.01	1.706	-1.233	0.0038	0.050
	W-O		1.743			
	W-O		1.787			
	W-O		1.792			
	W-O		2.166			
after 1 cycle in periodic operation test	W-O	0.84	1.753	8.669	0.0035	0.047
	W-O		1.790			
	W-O		1.836			
	W-O		1.841			
	W-O		2.225			
after reaction with electric field	W-O	0.88	1.709	-2.286	0.0040	0.014
	W-O		1.746			
	W-O		1.790			
	W-O		1.795			
	W-O		2.170			

[a] k- range: 3-11Å, R-range: 1-2.3 Å

Table S10 BET surface area of various catalysts as made and after reaction with electric field

Catalysts	BET surface area / $m^2\ g^{-1}$	
	as made	after reaction
$TBA\text{-}PW_{12}/CeO_2$	52.1	5.6
$Ce_2(WO_4)_3/CeO_2$	3.6	3.2
WO_3/CeO_2	90.1	18.6
$Ce_2(WO_4)_3$	1.0	0.7

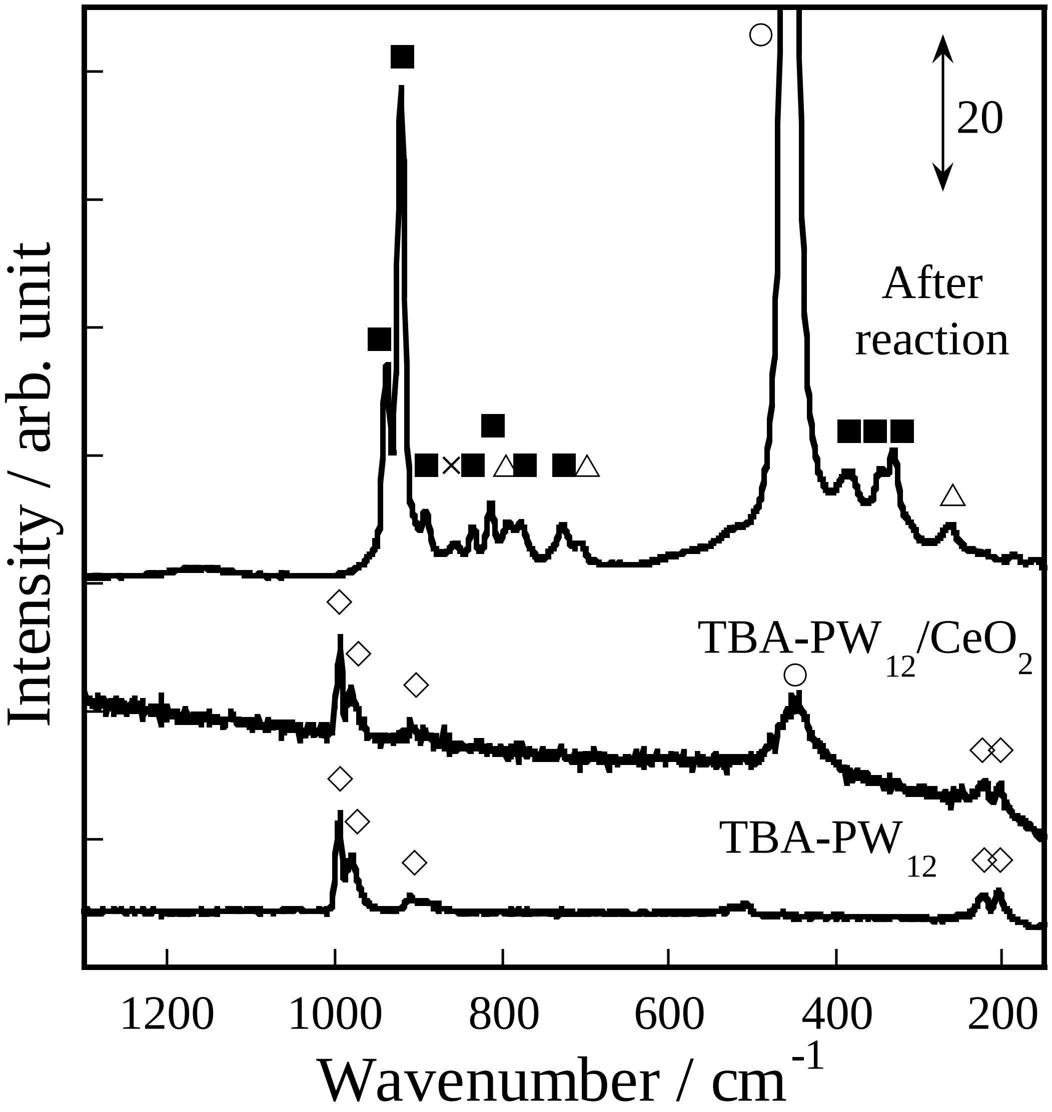

Fig. S1 Raman spectra of TBA-$PW_{12}O_{40}$ and 40 wt%TBA-$PW_{12}O_{40}$/CeO_2 before and after reaction with electric field.

○: CeO_2, ◇: $[PW_{12}O_{40}]^{3-}$, △: WO_3, ■: $Ce_2(WO_4)_3$, ×: Unidentified

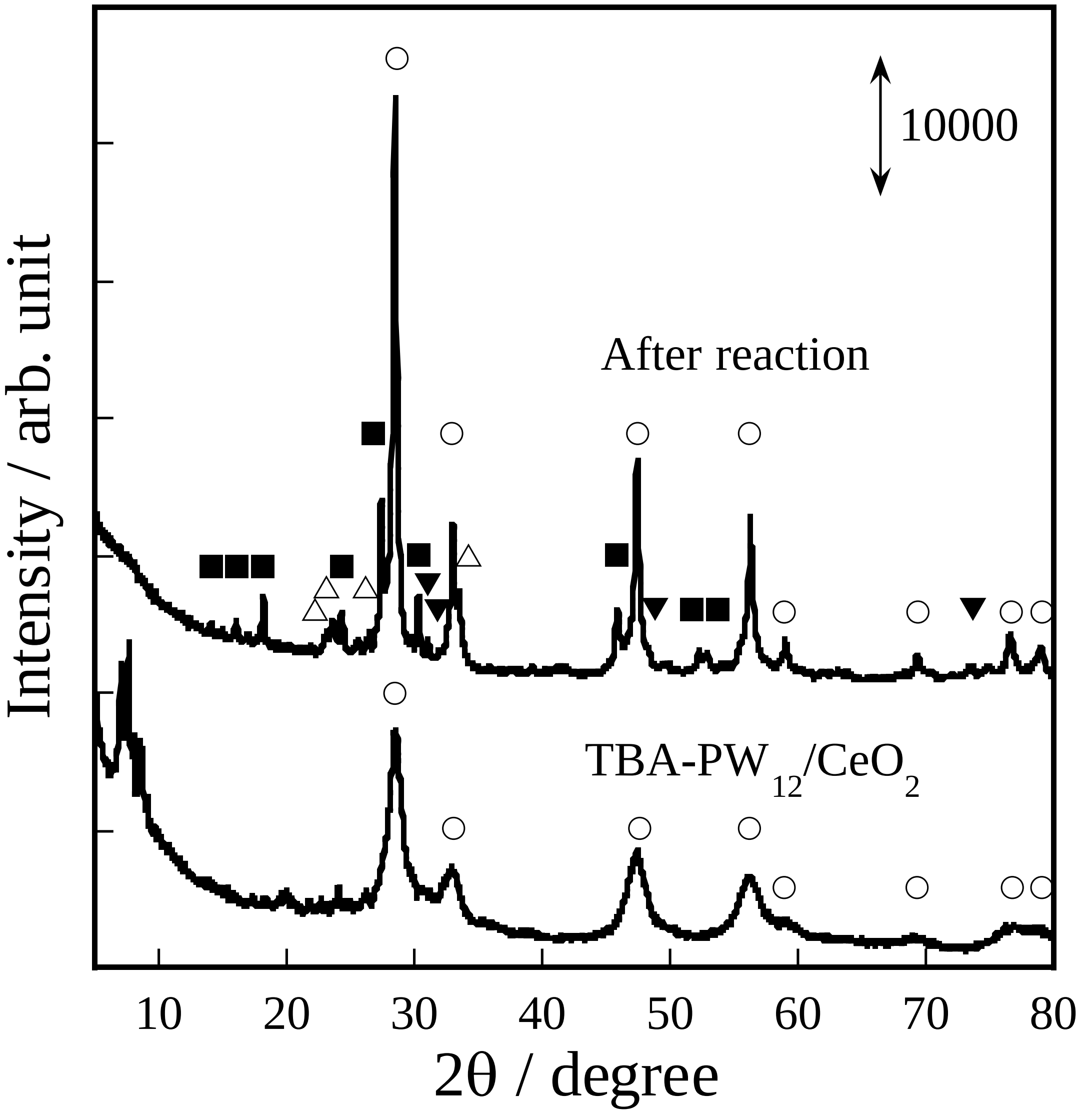

Fig. S2 XRD patterns of 40 wt%TBA-$PW_{12}O_{40}/CeO_2$ before and after reaction with electric field.

○: CeO_2, △: WO_3, ■: $Ce_2(WO_4)_3$, ▼: Ce_2WO_6

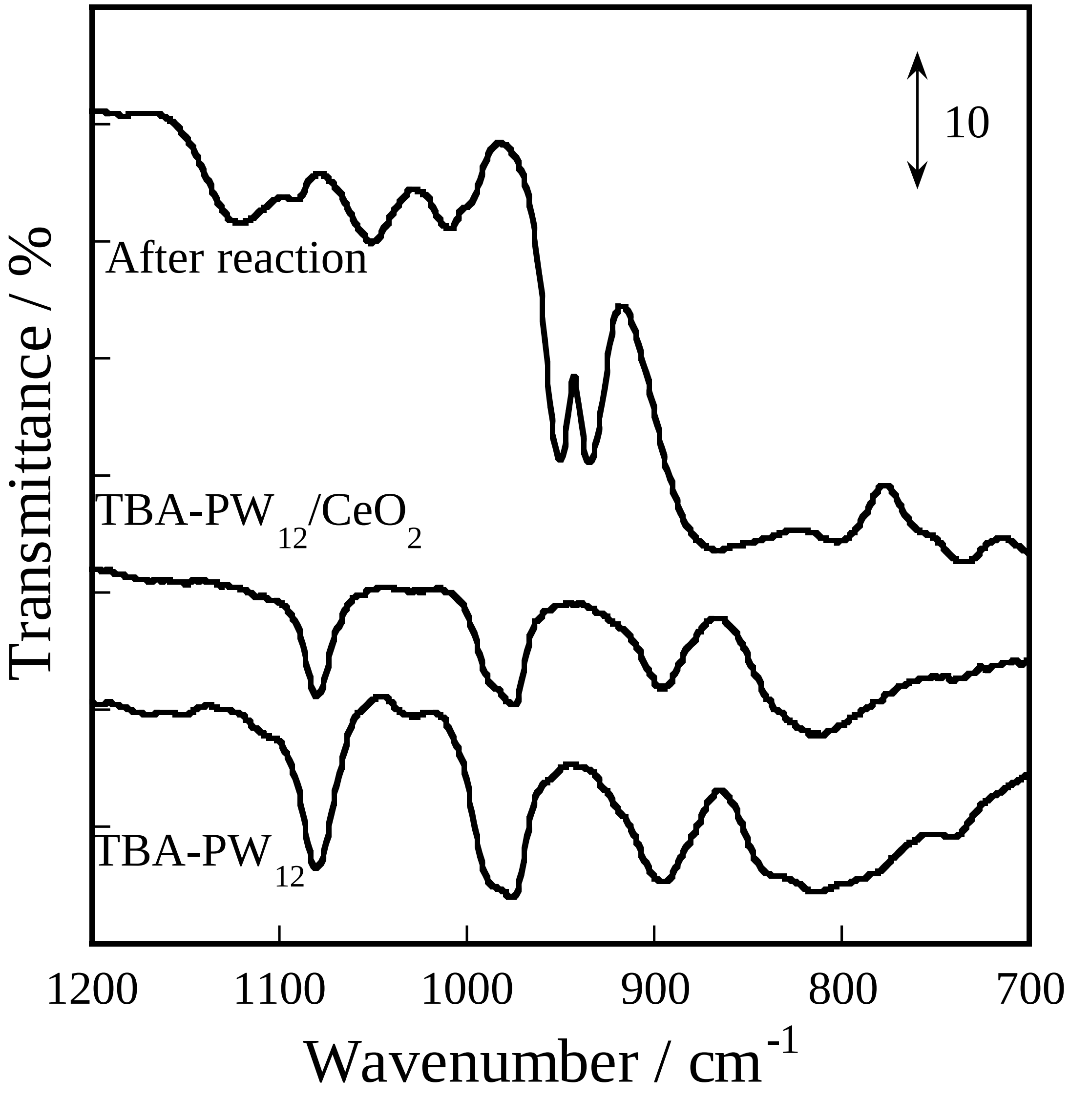

Fig. S3 IR spectra of TBA-$PW_{12}O_{40}$ and 40 wt%TBA-$PW_{12}O_{40}$/CeO_2 before and after reaction with electric field.

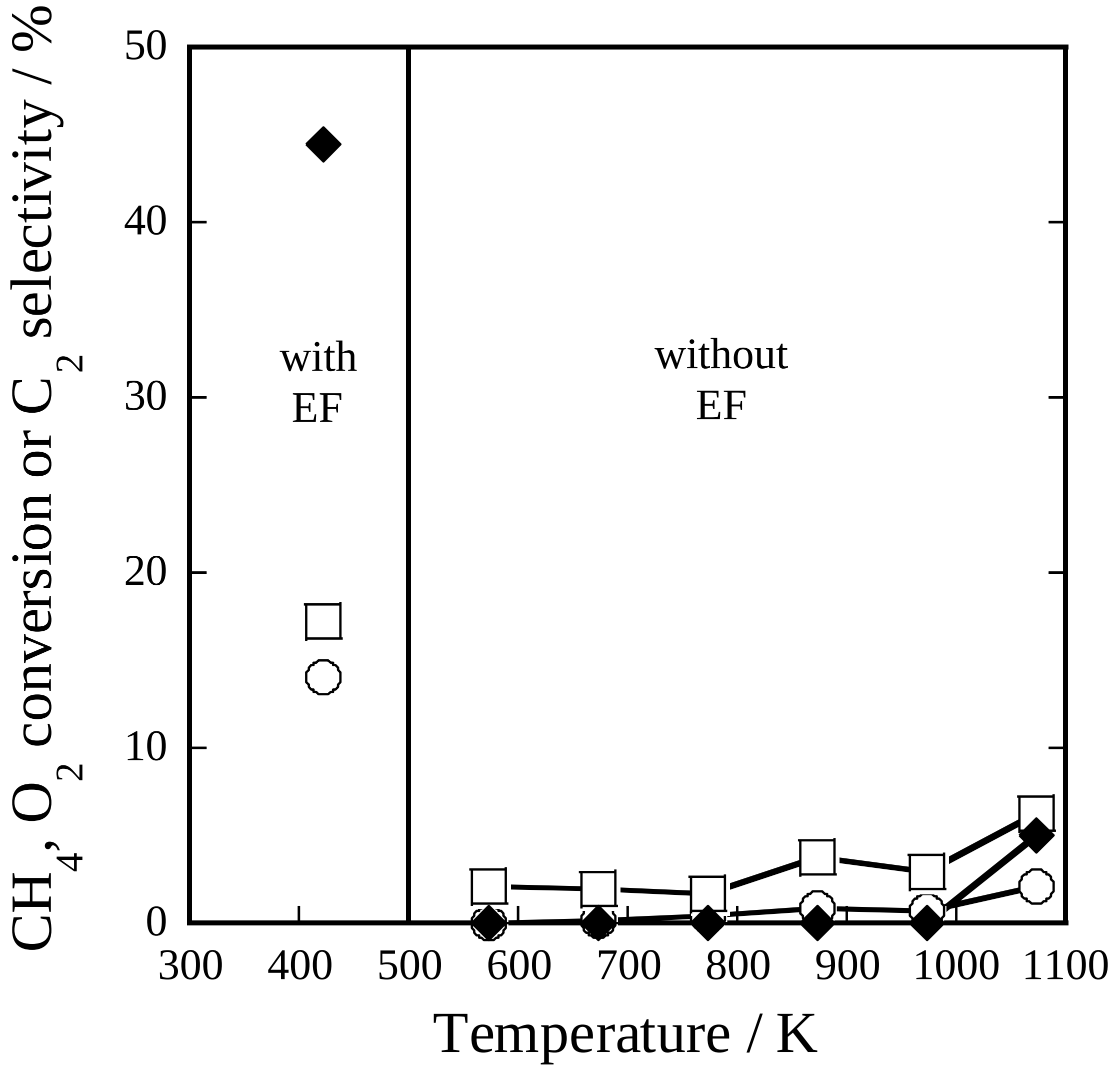

Fig. S4 Catalytic activity in the conventional reaction (573-1073 K) over 40 wt% TBA-$PW_{12}O_{40}/CeO_2$ after the reaction with electric field (423 K, 3.0 mA, 10 min). ○: CH_4 Conv., □: O_2 Conv., ◆: C_2 Sel.

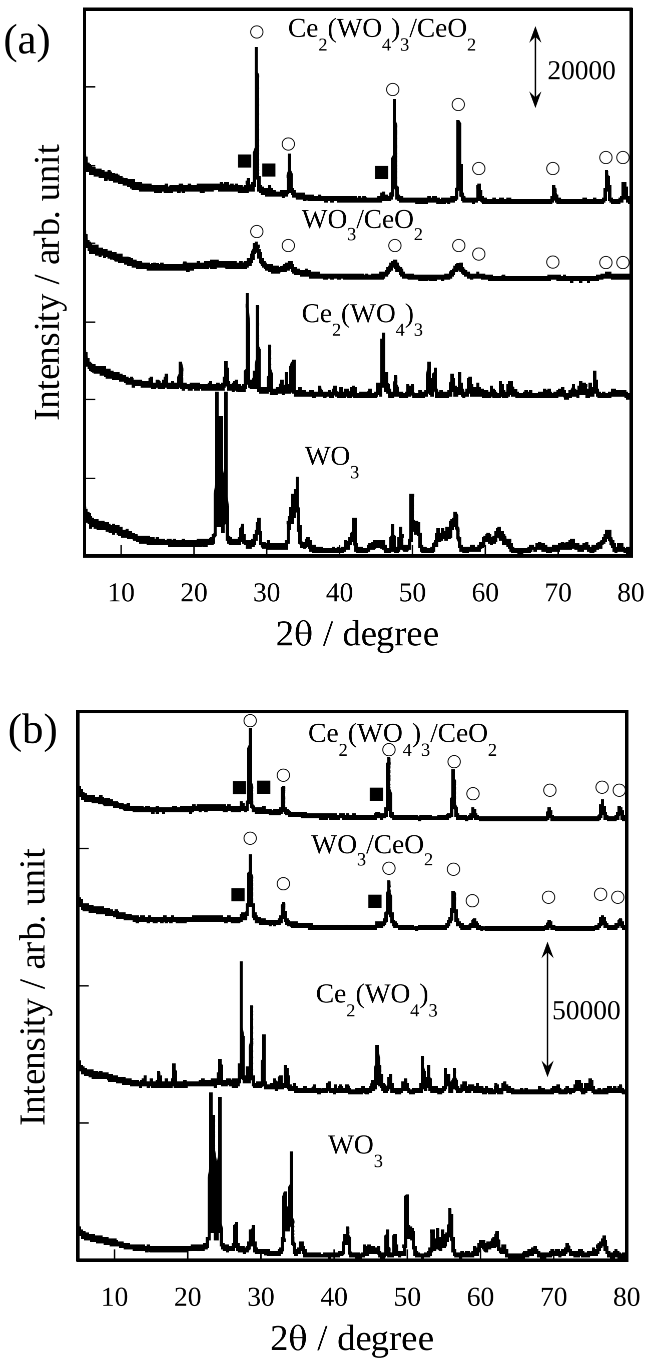

Fig. S5 XRD patterns of various oxide catalysts.
(a) as-made, (b) after reaction with electric field
○: CeO_2, ■: $Ce_2(WO_4)_3$

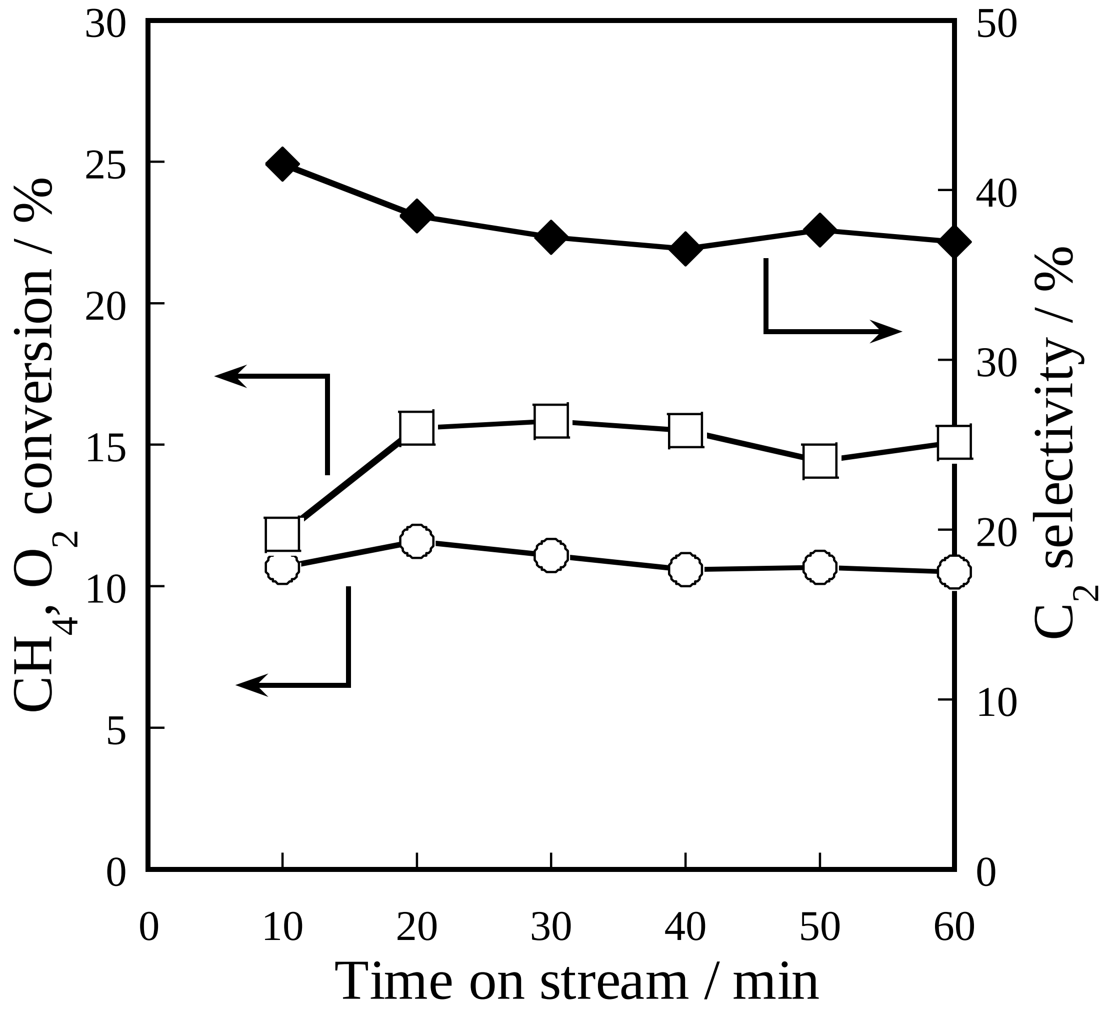

Fig. S6 Time course of the CH_4 and O_2 conversion and C_2 selectivity for OCM over $Ce_2(WO_4)_3/CeO_2$ in the electric field (423 K, 3.0 mA).

○: CH_4 Conv., □: O_2 Conv., ◆: C_2 Sel.

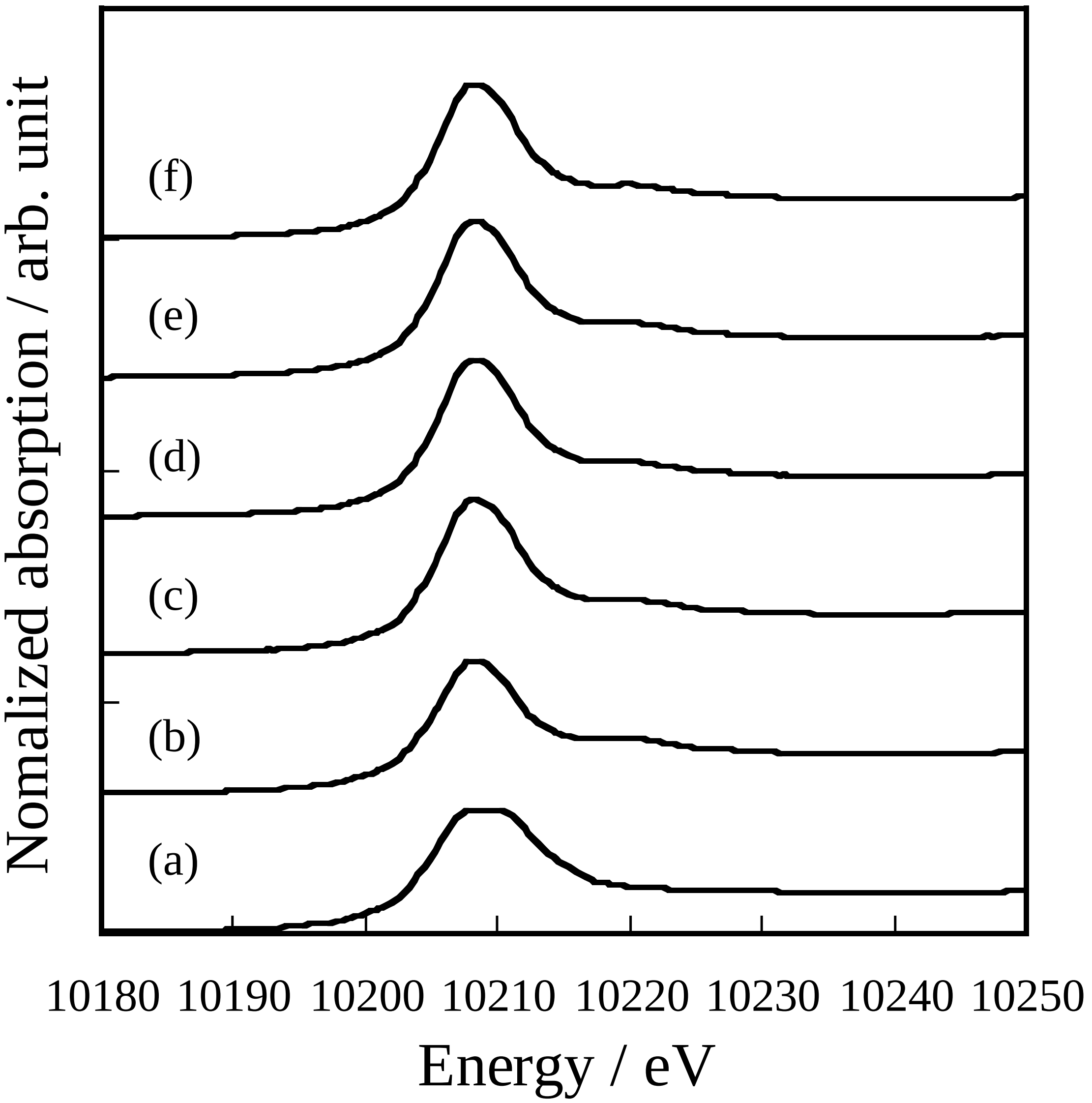

Fig. S7　XANES spectra at W L_3-edge over WO_3, $Ce_2(WO_4)_3$ and $Ce_2(WO_4)_3/CeO_2$ catalysts in various state.

(a) WO_3, (b) $Ce_2(WO_4)_3$, (c) $Ce_2(WO_4)_3/CeO_2$ as-made, (d) $Ce_2(WO_4)_3/CeO_2$ after O_2 supply in periodic operation test, (e) $Ce_2(WO_4)_3/CeO_2$ after 1 cycle in periodic operation test, (f) $Ce_2(WO_4)_3/CeO_2$ after reaction with electric field

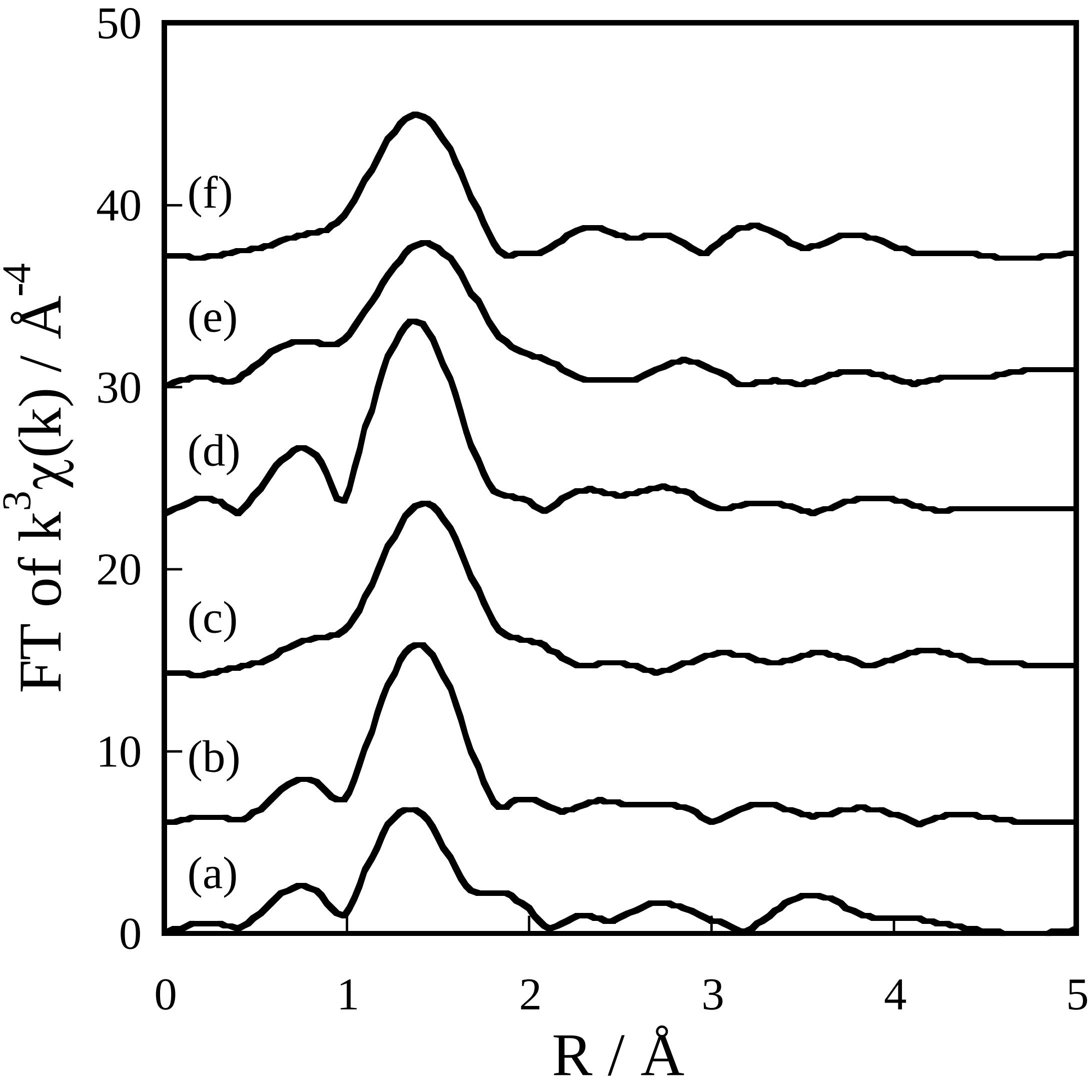

Fig. S8 EXAFS spectra at W L_3-edge over WO_3, $Ce_2(WO_4)_3$ and $Ce_2(WO_4)_3/CeO_2$ catalysts in various state.

(a) WO_3, (b) $Ce_2(WO_4)_3$, (c) $Ce_2(WO_4)_3/CeO_2$ as-made, (d) $Ce_2(WO_4)_3/CeO_2$ after O_2 supply in periodic operation test, (e) $Ce_2(WO_4)_3/CeO_2$ after 1 cycle in periodic operation test, (f) $Ce_2(WO_4)_3/CeO_2$ after reaction with electric field

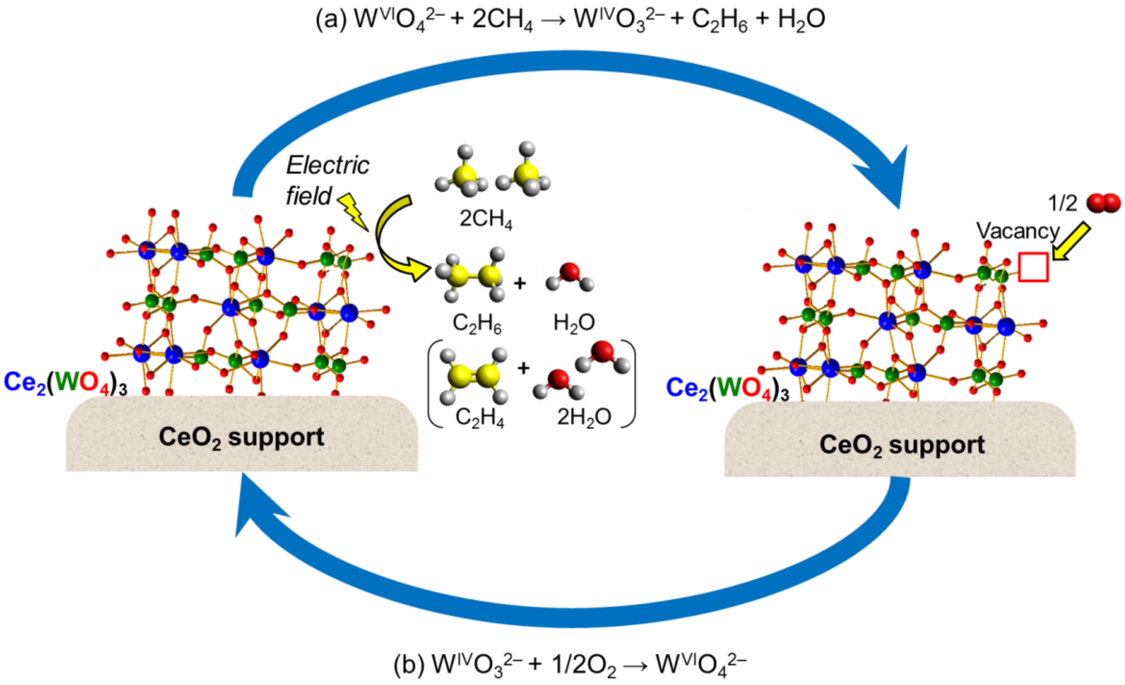

Fig. S9 Possible reaction mechanism of OCM over $Ce_2(WO_4)_3/CeO_2$ catalyst in the electric field:

(a) OCM occurred using lattice oxygen of $Ce_2(WO_4)_3$ (short W–O bond in distorted WO_4 unit); (b) Reproduction of lattice oxygen by gas-phase oxygen.

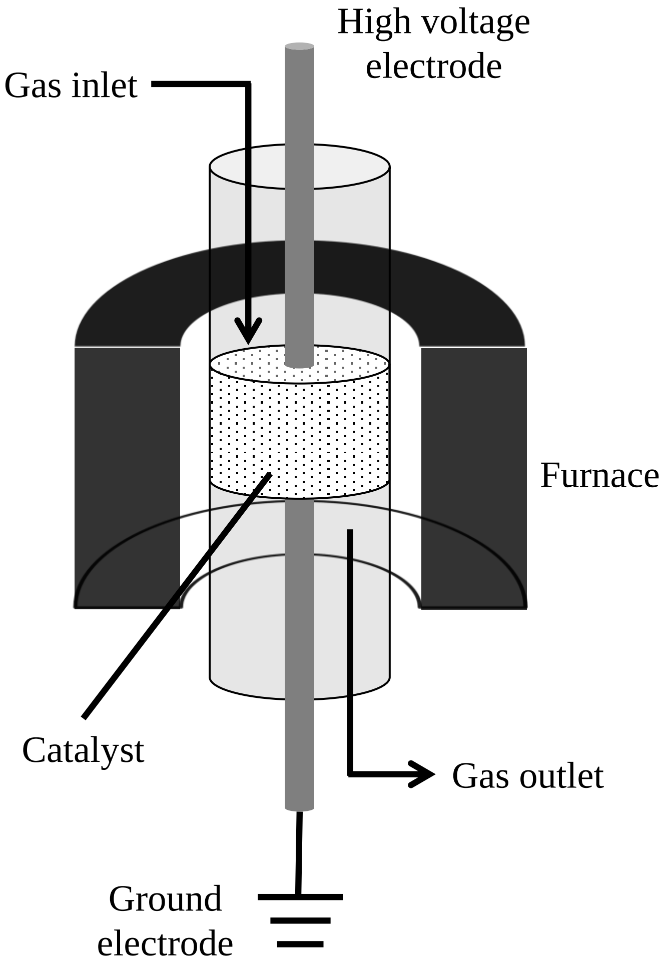

Fig. S10   Schematic diagram of reactor.

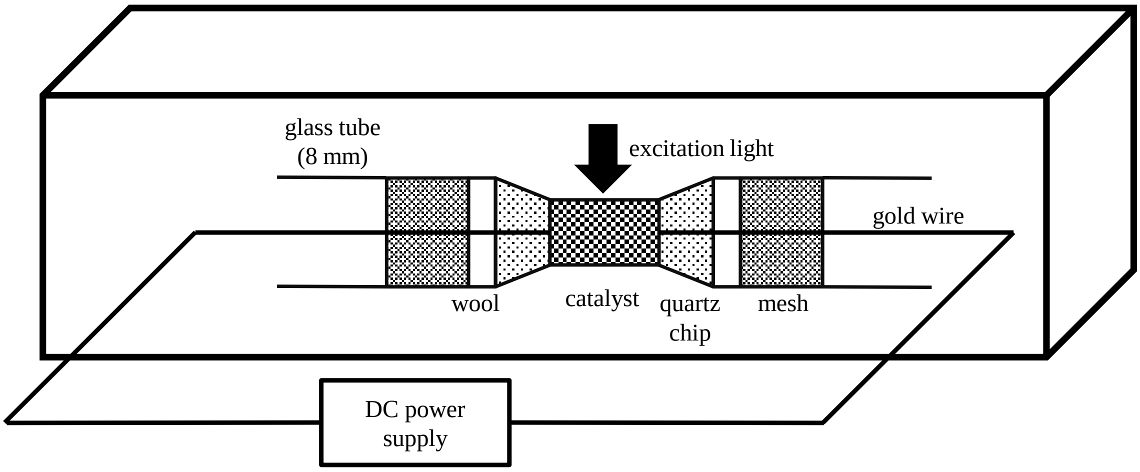

Fig. S11 Schematic diagram of reactor for *in-situ* Raman.
